# Examining the influencing factors and the functioning mechanism of online learning supporting learners’ empowerment

**DOI:** 10.3389/fpsyg.2022.1028264

**Published:** 2022-11-10

**Authors:** Xiaoquan Pan

**Affiliations:** Xingzhi College, Zhejiang Normal University, Jinhua, China

**Keywords:** online learning, environment elements, online behaviors, person factors, learners’ empowerment, reciprocal determinism, English language learning

## Abstract

The construction of an effective technology-based environment to promote learning and enable online learning to truly empower learners has become an increasingly important issue of concern. To some extent, an in-depth analysis of the key influencing factors and their functioning mechanism of online learning supporting learners’ empowerment may pave a new way for the understanding of this issue. In view of this, this study, grounded on the reciprocal determinism theory, explored the influencing paths among environment elements, online behaviors, person factors, and learners’ empowerment by using structural equation modeling (SEM) approach, and examined the structure effect relationships among these dimensions. A total of 396 Chinese university students reported on their perceptions of environment elements, online behaviors, person factors, and learners’ empowerment through the questionnaire survey. The results showed that there was a strong correlation between the three dimensions of the reciprocal determinism theory, and the three dimensions all exerted a positive effect on learners’ empowerment. Among them, the item parameter estimates revealed that online environment, online interaction, and online cooperation were significant in explaining the influence of environment elements and online behaviors on learners’ empowerment. Based on the empirical results, this study provided some implications and suggestions for the rationality of the structure devising of online learning environment, the effectiveness of online teacher-student interaction, and the development of students’ learning autonomy in technology-based learning. Thus, this study helped understand the specific contribution of external and internal factors to learners’ empowerment of using technology for online learning and would inform the construction of the optimized online learning environments and teacher-student relationships to enhance students’ sense of online presence and learning initiative.

## Introduction

With the rapid development of multimedia and Internet technologies and their wide application in distance education, online learning has emerged as a universally adopted way of learning in educational settings (Ellis and Bliuc, 2016). Online learning, conceptualized as using Internet technology to deliver learning experiences, is not a new concept (Moore et al., 2011). According to the systematic literature review from Zhang et al. (2022a), Singh and Thurman (2019) defined online learning as “education delivered in an environment through the use of the Internet for teaching content to enhance synchronous or asynchronous learning activities as well as learning that is not dependent on student physical or virtual location” (p: 622). In the narrow sense, online learning means that all participants in teaching and learning are online-connected and embedded in computer-mediated environment (McInnerney and Roberts, 2004); in the broad sense, online learning refers to all the teaching and learning activities conducted through computer networks, especially the Internet technologies (Panigrahi et al., 2018). Grounded on the full capitalization of the potential of technology for education, scholars depicted online learning as an activity that combines education with internet technology and ultimately generates or constructs new knowledge (Tzeng et al., 2007), and a process that disseminates and provides a complete set of knowledge resolutions through the Internet to create knowledge and improve its performance (Macgregor and Turner, 2009). Similarly, scholars’ understanding of superiority, accessibility, and flexibility of online learning has been embodied by an explicit assumption that online learning is a form of distance learning implemented by acquiring learning-related digital resources (Dublin, 2004) and promoted by the interactive learning environments through technologically mediated learning platforms (Ragusa and Crampton, 2017) and a technology-connected learning experience during which learners build and create knowledge (Lock et al., 2021). “Support for learning activity through technology” falls under the category of technology for learning (Margaryan et al., 2011) and therefore, online learning is essentially a technology-facilitated learning (Korhonen et al., 2019).

Flexibility as a feature of online learning might seem widely beneficial given its advantages of breaking through the time and space constraints of face-to-face courses, broadening access to learning resources, providing a convenient and flexible learning environment, and meanwhile supporting learners to develop the ability of collaborative learning, self-regulated learning, and critical thinking, but critiques of online learning have become quite common in the current literature in recent years, with a number of scholars attempting to examine potential barriers associated with online learning such as decrements in academic performance (Molnar et al., 2019), shallow interaction and surface learning (Barbour et al., 2018), feeling of isolation (Song et al., 2004), maladaptation to online environments (Yan et al., 2021), and lack of learning motivation (Muilenburg and Berge, 2005). Therefore, the construction of an effective technology-based environment to promote learning and enable online learning to truly empower learners has become an increasingly important issue of concern. To some extent, an in-depth analysis of the key influencing factors and their functioning mechanism of online learning supporting learners’ empowerment may pave a new way for the understanding of this issue. Although there have been studies examining the interrelationship between learners’ behavioral outcomes and the specific implementation of the online learning technological artifact, such as learner satisfaction (Sun et al., 2008), online learning success (Kauffman, 2015), and technology-based academic performance (Yates et al., 2020), these approaches did not account for the specific relational nature of subjectivity which may open a pathway into far more multivariate forms of conducting research with the regard to learner characteristics, especially learners’ empowerment. By combing the existing literature, it can be found that the reciprocal determinism developed by the American psychologist Bandura (2001) is acclaimed for constituting the theoretical foundation for the study of online learning. According to this theory, individual behavior intentions are formed by the interaction of three key psycho-socio components: environment, person, and behavior. Furthermore, the three components are mutually interrelated and determined, thus constituting a complete system. As such, the reciprocal determinism transcends the traditional “one-way determinism” of cognitive psychology and takes into account a three-dimensional framework of environment, person, and behavior, thus building a bridge between the individual’s internal cognition and external environmental interaction (Dörnyei and Ushioda, 2009; Chi et al., 2017). Based on reciprocal determinism, current literature has explored the result of the interaction between individual factors and the external environment, such as learning engagement (Pan and Shao, 2020), learning attitude toward online learning (Chen and Yu, 2019), perceived educational potentials of technology (Lai, 2015), and perceived compatibility between technology use and learning expectancies (McLoughlin and Lee, 2010). However, an important variable that is closely intertwined with the fundamental driving force on technology use for online learning—learners’ empowerment (Luechauer and Shulman, 1993; Frymier et al., 1996)—remains less explored. By building upon the aforementioned relevant works, this study aimed to contribute to the online learning literature with a comprehensive analysis of the influencing factors and functioning mechanism of online learning supporting learners’ empowerment, with the expectation to providing an empirical basis for the research and design of online learning.

## Literature review

### The concept and structure of learners’ empowerment

In fact, as for the research of online learning, the comparison of the discrepancy between online and offline learning and the analysis of students’ sense of experience in online learning will be the future orientation (Arbaugh, 2004). This category of research actually attempts to answer the question of whether the online learning is really effective, which impels people to concern on the effectiveness of online learning (Arbaugh et al., 2009). Scholars have conducted related research from diverse aspects, such as exploring college students’ mobile learning continuance from an online-*cum*-offline learning perspectives (Zhang et al., 2022b); considering students’ intrinsic motivation and internally psychological needs as important influencing factors of online learning (Chen and Jang, 2010); thinking of a good personalized virtual environment being of positive significance to improve the effectiveness of students’ online learning (Xu et al., 2014). As such, to explore online learning supporting learners’ empowerment will be a new perspective of study, that is, whether online learning can promote learners' value identity and self-reinforcement can be further examined.

The concept of “empowerment” was originally closely related to “Power,” and typically referred to “resistance to oppression and the pursuit of justice” (Zimmerman, 2000). After decades of development, it is now widely used in the economic, political, and educational fields, and has brought about broader connotations, usually involving increased participation, empowerment, autonomy, and decision-making (Hur, 2006). Scholars have given different definitions of empowerment. For instance, Byham et al. (1992) narrowly defined “empowerment” as the capacity of learners to make decisions, the process of holding oneself accountable for their learning, while Luechauer and Shulman (1993) broadly defined it as “the humanistic process of adopting the values and practicing the behaviors of enlightened self-interest so that personal and organizational goals may be aligned in a way that promotes growth, learning, and fulfillment” (p: 13). In the field of education, learners’ empowerment refers to the process by which learners accomplish tasks, improve self-efficacy, and develop multiple capabilities in specific learning situations and activities, which focuses on the transformation of learners’ thinking, the process of identifying with task values, and the reinforcement of self-meaningful behaviors, thus making individual and collective goals consistent so as to ultimately promote the realization of collective goals (Frymier et al., 1996).

Existing studies of technology-based language learning have generally shown a close relationship between learning environment and learners’ empowerment. Learning environment is considered to be one of the antecedents of constructing the basis of students’ online learning identity and a “wider ecology of learning” (Sefton-Green, 2006, p: 4). Thus, considering the significant association between online learning environments and learners’ empowerment, it is important to understand how language learners are utilizing technology-enhanced environments to develop their self-development capabilities. The technology-based learning environment gives learners more options to tap and stimulate their individual potential. When learning is self-directed and initiated by students of their own choice, the passion for active participation in learning is stimulated. Therefore, the environmental empowerment education promotes learners’ self-improvement and multivariate development.

Grounded on the existing literature (Frymier et al., 1996; Yang et al., 2020), learners’ empowerment in this study was described in terms of two key dimensions: (1) *value identification*, which focuses on learners’ identity to the meaning and value of learning in relation to one’s own beliefs, ideals, and standards; and (2) *self-reinforcement*, which refers to learners’ engaging in self-fulfilling behaviors in relation to the individual’s perception of their own learning capability. According to Frymier et al. (1996), as learners perceive of their capability of completing a learning task, their sense of empowerment is increased and they become more confident in learning, and when individuals lack confidence or feel unqualified, their sense of empowerment is diminished. Thus, the sense of empowerment plays a key role in learners’ learning and development (Yang et al., 2020). Considering that pedagogical guidance exerts a profound impact on students, it is necessary to enable students to have a sense of empowerment which embodies the internalization of positive attitudes that ultimately results in a heightened sense of personal effectiveness.

### The influencing factors of online learning supporting learners’ empowerment

Online learning supporting learners’ empowerment signifies espoused beliefs of what is deemed important to the technology adoption for self-regulated learning at the individual level, involves the multivariate predicting factors for online interaction through which learners act and communicate, and thus is considered to be powerful explanation of the interrelations between external environments, learners’ internal influencing mechanisms (e.g., motivation and attitude), and learning behavioral intentions (Sadeghi and Kardan, 2015; Lai et al., 2016; Guo, 2018). This fits in with the three-dimensional reciprocal determinism put forward by Bandura (2001). What is unique about this theory is that it distinguishes between human behavior and cognitive factors, pointing out the role of cognitive factors in determining behavior. Based on the reciprocal determinism, this study combined the characteristics of university students’ online learning with the existing technological environments, and analyzed three important dimensions (environment elements, online behaviors, and person factors) of online learning that affect learners’ empowerment.

#### Environment elements

This study used online environment and resource support to characterize environment elements. Firstly, online environment is a multidimensional variable, involving the effective construction and coordination of learning situation, learning activities, and teaching design (Fraser, 2012). Learning situation is a comprehensive description of one or a series of learning events or learning activities under which learning occurs. Learning activities refer to the sum of the actions taken by learners and their related learning groups (including peers and teachers, etc.) to achieve a specific learning goal (Beetham and Sharpe, 2007), and highlight the assumption of emotional and intellectual responsibilities in learning. Teaching design is the assumption and plan that arranges the elements of instruction and determines the appropriate teaching scheme. In the context of technology use for online learning, helping organize learners to communicate and cooperate in learning activities with various techniques, tools, and means and enabling them to access and make effective use of these resources to promote knowledge construction are essential (Lai, 2015). Online learning as a technology-facilitated learning should be based on the learning theories and pedagogy that are appropriate to it (Kop and Fournier, 2011), with its effectiveness building largely on how the technology is designed and implemented in teaching and learning (Johnson, 2017; Eynon and Malmberg, 2020). Teachers have a big role to play in choosing teaching strategies to ensure the integration of students’ knowledge acquisition and their own learning experience in online environments (Ertmer and Ottenbreit-Leftwich, 2010), and to accelerate the understanding of the basic characteristics of technology to enhance learning. Secondly, resource support is the basis of online learning support, characterized as a facilitation condition which is thought to help enhance learners’ sense of community, belonging, identity, and knowledge sharing in the process of online learning (Hsu et al., 2012; Peñarroja et al., 2019). In the technology age, learners’ knowledge sources are no longer confined to the teaching materials offered by teachers, for learners can seek the diversified digital learning materials with the help of technology to broaden their horizons. The existing literature has long emphasized the significance of resource support for the effective implementation of students’ online learning. Considering that abundant learning resources are the guarantee of online learning, Wang (2007) conducted an empirical research on what autonomous language learners expected their teachers to do, finding that learners rated teachers’ roles in providing resources and learning strategies as more important than other roles. Similarly, Lai (2015) found that resource support exerted a positive impact on learners’ empowerment, that is, the recommendation of useful technical resources, the guidance and help of using these resources efficiently, and the encouragement of promoting students to use these resources. Research evidence also suggested that the availability of online learning resources can stimulate interest and curiosity among learners and promote understanding and efficient inquiry (Mamun et al., 2020).

#### Online behaviors

In this study, online behavior is mainly characterized as online interaction, online collaboration, and resource acquisition. Online learning has the characteristics of interaction, sociality, and cognitive presence, thus resulting in a stronger emphasis on sharing experience than in traditional settings (Abedini et al., 2021). A review of the research on distance online learning shows that the key role of interaction in online learning has been long well-recognized by scholars. For instance, Garrison and Anderson (2011) argued that the increasing opportunity for interaction between teachers and students, as well as between learners, is one of the key factors in the success of distance learning. According to Woo and Reeves (2008), instructional interaction is the most important element in the design of online learning. Acknowledging the critical role of interaction, Trentin (2000) proposed that the quality of online learning depends on instructional interaction and Kumi and Sabherwal (2018) argued that the lifeline of online learning is the deep interaction that points to the realization of multidimensional connectivity. Interaction is also the core of collaborative learning. As a category of learning mediated by social activities, online collaborative learning is an important way to cultivate the basic quality and ability of citizens in the 21st century, such as cooperation, collaboration, and innovation. Extant literature suggested that collaborative learning can stimulate learning enthusiasm, make students more active in learning, increase sense of responsibility for learning, enhance the ability of cooperation, improve learning efficiency, and then promote their academic performance (Kumi and Sabherwal, 2018; Panigrahi et al., 2018). In the online learning environment, the utilization of technical tools provides the possibility to achieve the multidimensional interactive collaborations between teachers, students and human-computer components, thus providing a new chance for knowledge creation. In addition, resource acquisition constitutes an important component of online behaviors. Faced with massive, extensive, and fragmented online learning resources, college students are prone to knowledge maze. Therefore, effective access to learning resources has become the key competence of learners’ empowerment to enhance university students’ self-reinforcement in online learning, as digital competences are highlighted as a set of knowledge, skills, attitudes (thus including abilities, strategies, values, and awareness) that are required when using ICT and digital media to perform tasks; solve problems; communicate; manage information; collaborate; create and share content; and build knowledge effectively, efficiently, appropriately, critically, creatively, autonomously, flexibly, ethically, reflectively for work, leisure, participation, learning, socializing, consuming, and empowerment (Avila et al., 2020).

#### Person factors

Person factors mainly involve self-induced factors such as learning motivation and self-evaluation ability. Learning motivation is the sum of the incentives that positively force the choice of a specific behavior or purpose (Jarvis, 2005) and is intrinsically driven and self-motivated, which was confirmed to be still crucial for foreign language learning (García-Sánchez and Luján-García, 2016). Research evidence showed that learning motivation had a strong promoting effect on cognitive process of online learning, involving cognitive strategy use and self-regulation (Pintrich and Schunk, 2002; Zusho et al., 2003) and that learning motivation functioned as a mediating mechanism illustrating relations between students’ perception of technology environments and their attitude toward technology-based self-directed learning (Pan, 2020). This implies that students’ online learning behavioral intentions can be different depending on their motivational facets, namely motivation influences students’ cognitive strategy use and self-regulation by facilitating or impeding self-monitoring processes (Pekrun, 2006). Self-evaluation is viewed by Bransford et al. (1999) as “people’s abilities to predict their performances on various tasks and to monitor their current levels of mastery and understanding” (p: 12). In the online learning context, self-evaluation of learners is characterized as the ability of setting goals, regulating their own learning process, using appropriate learning strategies, and making efforts to achieve the predetermined learning goals. This kind of self-evaluation has the extremely vital significance to the online learning behavior empowerment, and has evidenced to have a significant correlation with students’ online learning behavior and as well to positively predict students’ motivational learning outcomes, such as attitudes toward learning tasks and academic self-efficacy (Kormos and Csizer, 2014; Cho et al., 2017). This signifies that students with higher self-evaluation are more likely to adopt well-planned learning methods, to map out learning time, to be autonomously aware of learning outcomes and process, and to actively create the physical and social environment conducive to learning (Klenowski, 1995). Students with strong self-evaluation capability are more aware of the value of online learning, will actively set learning goals and quickly enter the learning state (Cho et al., 2017), and can maintain learning motivation to obtain better online learning experience (Akyol and Garrison, 2011).

## Methodology

### Research questions and hypothesis model

Informed by recent new visions in the study of online learning supporting learners’ empowerment above, two research questions are specified below.

1. In online learning, what level of perception do students have about environment elements, online behaviors, and person factors?
2. In online learning, do environment elements, online behaviors, and person factors affect learners’ empowerment, and what is the functioning mechanism?

In line with these two research questions, the following factors and related associations are highlighted to affect online learning supporting learners’ empowerment, and thereby research hypotheses were generated.

The three-dimensional reciprocal determinism put forward by Bandura (2001) conceptualized environment, behavior, and person factors as mutually determining elements, concentrated on the influence of human behavior and its cognitive factors on the environment, and averted the tendency of mechanical environmental behaviorism. Combined with existing literature, this study put forward the following hypotheses (H1–H3).

*H1*: Environment elements correlate with online behaviors.

*H2*: Online behaviors correlate with person factors.

*H3*: Environment elements correlate with person factors.

The technology-based online environment provides students with a convenient and practical way to connect with the construction of human knowledge (Sawyer, 2014) by utilizing a variety of technological tools to increase knowledge-sharing opportunities and engage in collaborative activities (Jesionkowska, 2020). Previous research found the importance of learning environment elements in predicting the construction of students’ online learning identity (So and Brush, 2008). With technologies enabling the environment, the construction of good learning environment entails the support of technology, which endows learners with the power of free choice and increases the opportunity of learning engagement, thus making the learning environment more suitable for the independent development of learners. The educational achievement builds on the learning environment which accelerates students’ omni-directional development as the core, strengthens the sense of social responsibility, and improves the capacity of self-regulation (Pintrich, 2000). Therefore, this study hypothesized that:

*H4*: Environment elements significantly and positively influence learners’ empowerment.

Online learning utilizes social networks to build online communities of practice which help mitigate the hinderers of distance, time limitation, and members’ isolation and increase opportunities for knowledge sharing, engaging in collaborative activities, and sustaining these interactions (Jesionkowska, 2020). In addition to this, existing research acknowledged that online behaviors from online communities of practice function as a catalyst for improving students’ engagement (O’Neill et al., 2018), stimulate the students’ information exchange if they share a common understanding and skill (Kumi and Sabherwal, 2018), and reach the conceptual level of collaborative knowledge construction process (Ioannou et al., 2015). These components of online learning behavior are conducive to stimulating students’ identity and perception of their own learning capability, thereby promoting learners’ empowerment. Hence, this study hypothesized that:

*H5*: Online behaviors significantly and positively influence learners’ empowerment.

From the psychological perspective of individual motivation, “empowerment” is to control the allocation of resources by meeting the internal development needs of the individual, so as to improve individual efficiency and enhance the individual’s motivation to achieve goals (Zimmerman, 2000). With learners’ empowerment in education, learners possess more autonomy, participation, and developmental initiatives, forming a “learner-centered” pattern in teaching and learning (Yang et al., 2020). On the one hand, learners have access to the online learning network environment, share, collaborate, and communicate in interactive communication, and achieve a sense of group belonging; on the other hand, learners’ empowerment helps control, participate, and use learning resources, and promote adaptation and self-development with a positive and active learning attitude. Individual’s unique psychological traits such as learning motivation and self-efficacy will affect their empowerment. Thereby, this study hypothesized that:

*H6*: Person factors significantly and positively influence learners’ empowerment.

**Figure 1:**
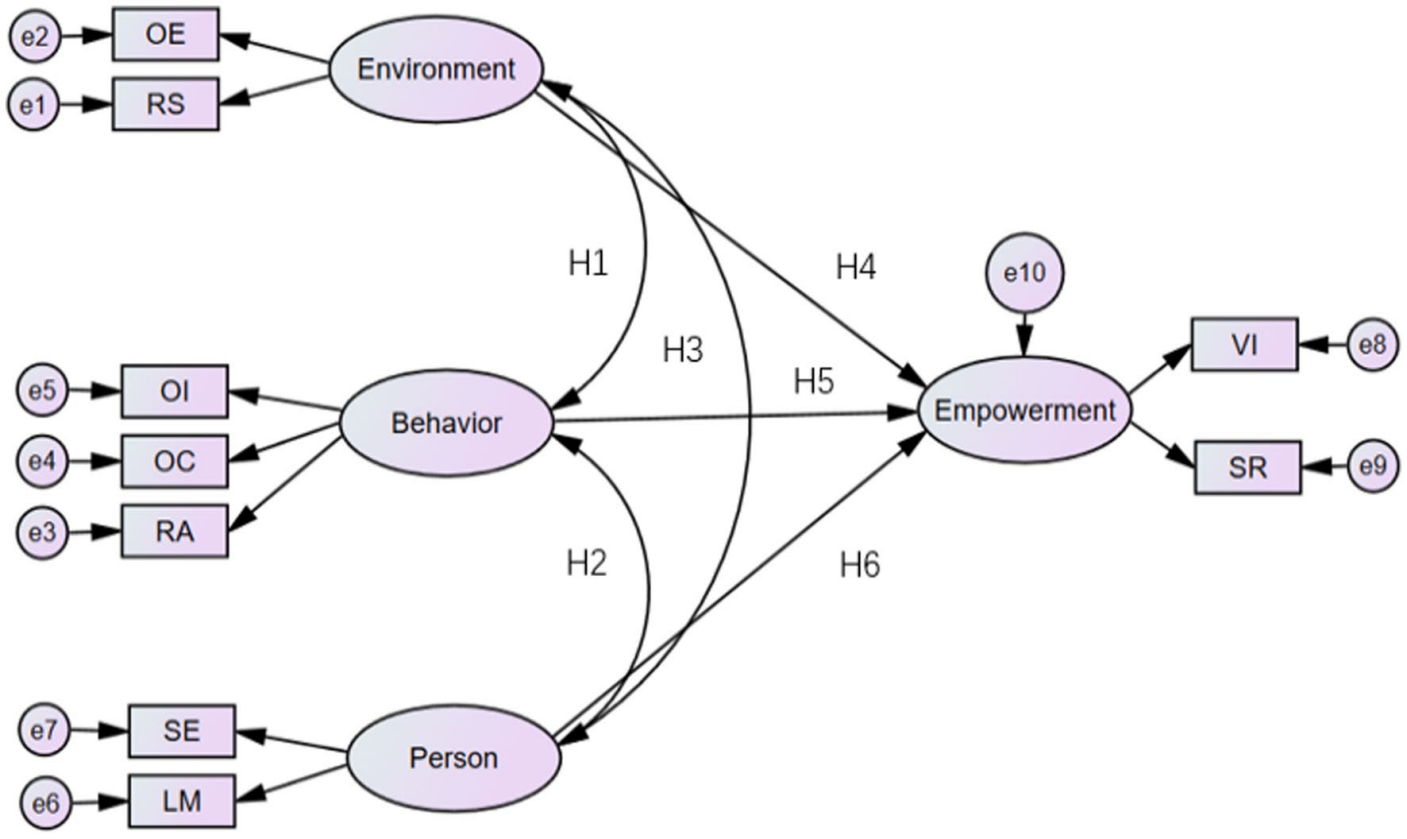
Research hypothesis model.

The following hypothesis model (Figure 1) corresponded to the above two research questions and six hypotheses.

### Participants and procedure

Participants were 405 undergraduates from a comprehensive university in Eastern China who were taking a college English course for the third semester. In China, college English, as a compulsory course for undergraduate students for a minimum of 2 years, involves the exclusive use of English as a second language as the medium for instruction and learning. With the wide application of technology in college English teaching, the teaching and research group of this course in this university adopted the blended teaching mode and combined traditional face-to-face classroom instruction with online teaching *via* a network platform, where teachers and students interact, collaborate, manage information, share and create knowledge, and conduct feedback. Therefore, students have possessed online learning experiences, which constitutes the favorable fundamental condition to conduct this research.

At class intervals, 405 participants completed a face-to-face questionnaire survey within about 10 min, with 396 (130 males, accounting for 32.83%) providing valid responses. The survey was collected immediately after completion. It is worth mentioning that participants were selected from the parallel classes with a view to achieve a representative sample in this study. They were briefed on the purpose of this study and told of their rights to withhold their participation during or after they had completed the questionnaire. As all students participated in the online learning through the network platform and approved of informed consent, their participation in the survey was cooperative and voluntary. To avoid potential bias, the survey was conducted anonymously.

### Measures

This research adopted the form of questionnaire to carry out this survey. The questionnaire comprised two parts. The first part consisted of basic information, involving age, gender, and the weekly duration of technology-based online learning. The second part consisted of four subscales, including environment elements, online behaviors, person factors, and learners’ empowerment, with a total of 41 items. The items of the above subscales all employed Likert 6-point scale, with 1 representing “strongly disagree” to 6 representing “strongly agree.” The higher score illustrated the higher level of perception of the corresponding item.

#### Environment elements

This dimension examined the influence of environment elements on online learning supporting learners’ empowerment with 10 items, involving online environment (5 items, α = 0.927) and resource support (5 items, α = 0.923). The devising of related items mainly referred to the distance education learning environment survey (DELES) scale developed by Walker and Fraser (2005). A sample item is “I think the teacher could provide effective online learning materials for my learning.” The standardized factor loadings (SFLs) of the 10 items ranged from 0.844 to 0.917, and the Cronbach’s α value and the Kaiser-Meyer-Olkin (KMO) value for validity were 0.950 and 0.914, respectively, indicating that the scale had good reliability and validity.

#### Online behaviors

Sun et al. (2008)’s interaction scale, which was widely used in the field of online learning research due to its high reliability and validity and was typically utilized to evaluate the interactive experience of online learning and to guide the design of online learning environment, was adopted and revised to fit this study. The revised scale consisted of three subscales: online interaction (4 items, α = 0.881), online collaboration (5 items, α = 0.932), and resource acquisition (3 items, α = 0.852). Participants rated the degree of conformity with their actual online behaviors. A sample item is “I was willing to conduct collaborative learning on the online platform.” The SFLs of the 12 items ranged from 0.812 to 0.914. On the whole, the Cronbach’s α value was 0.945, and the KMO value was 0.931, indicating good reliability and validity.

#### Person factors

This dimension consisted of self-evaluation and learning motivation. The self-evaluation scale (4 items, α = 0.852) was adapted from the online self-regulated learning scale developed by Barnard et al. (2009). The scale was widely used in educational technology, online learning, and other fields, and was assessed to have good reliability and validity. A sample item is “I think I knew how to use technological resources or tools through online platform.” Besides, the scale of online learning motivation (5 items, α = 0.941) was adapted and revised from Guilloteaux and Dörnyei’s (2008) and Kormos and Csizer (2014) studies to fit the study context. A sample item is “I enjoyed learning English through online platform.” The SFLs of the 9 items ranged from 0.718 to 0.936. On the whole, the Cronbach’s α value was 0.917, and the KMO value for validity was 0.891, indicating that the whole scale had good reliability and validity.

#### Learners’ empowerment

Combined with the research practice, the learners’ empowerment scale was adapted from the related research from Frymier et al. (1996) which fundamentally highlighted an increasing internalization of positive attitudes toward the content or subject matter, and a cognitive belief state of personal involvement that ultimately results in a heightened sense of personal effectiveness. The wording of items was modified for the current study so that items were anchored to a university context (e.g., “I think I felt confident in conducting online collaborative learning with peers”). This scale consisted of value identification (5 items, α = 0.920) and self-reinforcement (5 items, α = 0.936). The SFLs of the 10 items ranged from 0.839 to 0.924. The total Cronbach’s α value was 0.952 and KMO value was 0.946, which indicated that the scale had good reliability.

### Data analysis

This study employed SPSS 21.0 to conduct descriptive analysis and correlational analysis on the perceived level of learners’ empowerment and its influencing variables. Then, the structural equation modeling (SEM) approach was used to analyze the collected data. “Structural equation modeling (SEM) was employed in this study for its ability to analyze relationships between latent and observed variables” (Teo and Noyes, 2014, p: 2435). The standard two-step approach to SEM (Schumacker and Lomax, 2010) was adopted, with the first phase of estimating the measurement model for all latent variables in the model, which describes how well the observed indicators measure the unobserved (latent) variables, and the second step of estimating the structural part of the SEM, which specifies the relationships among the exogenous and endogenous latent variables.

## Results

### Demographic information and descriptive statistics

In the demographic descriptions in the questionnaire, the mean age of the participants was 18.521 (SD = 0.565) years, and the duration of technology-based online learning was specifically reported into learning when interested (53 students, accounting for 13.38%), less than 2 h per week (92 students, accounting for 23.23%), 3 to 6 h per week (165 students, accounting for 41.67%), and more than 7 h per week (86 students, accounting for 21.72%).

The descriptive statistics and correlational matrix of variables are shown in Table 1. As can be seen, students had a high level of perception of learners’ empowerment and its influencing factors, with an average of over 4.2, which was close to the option of “agree.” It can be found that in the process of online learning, learners had a strong sense of empowerment, a high sense of identification with environment elements, online behaviors, and person factors, and a high sense of self-reinforcement, but the perception of value identification was relatively low.

**Table 1:** Descriptive statistics and results of the measurement model.

Dimensions	Variables	Number of items	SFLs	Mean	SD	AVE	CR
Environment	OE	5	0.860–0.917	4.544	0.976	0.776	0.945
	RS	5	0.844–0.890	4.698	0.887	0.766	0.942
Behavior	OI	4	0.812–0.896	4.371	0.965	0.739	0.919
	OC	5	0.862–0.914	4.344	0.972	0.790	0.950
	RA	3	0.861–0.894	4.636	0.883	0.772	0.910
Person	SE	4	0.718–0.881	4.318	0.994	0.699	0.902
	LM	5	0.852–0.936	4.460	0.998	0.810	0.955
Empowerment	VI	5	0.839–0.904	4.251	0.992	0.758	0.940
	SR	5	0.840–0.924	4.427	0.969	0.801	0.953

N = 396; OE = online environment; RS = resource support; OI = online interaction; OC = online collaboration; RA = resource acquisition; SE = self-evaluation; LM = learning motivation; VI = value identification; SR = self-reinforcement.

### The reliability and validity of the questionnaire

In this study, the Cronbach’s α coefficient was used to test the reliability of the questionnaire. After calculation, the Cronbach’s α coefficient of each dimension was greater than 0.8, indicating that the internal consistency of the questionnaire structure was good, the results were reliable and had strong explanatory power. In terms of reliability and validity of the survey, this study took two steps: (1) the reliability analysis was employed. The results showed that the KMO values of environment elements, online behavior, person factors, and learners’ empowerment were all greater than 0.8, the results of Bartlett’s spherical test were all less than 0.05, which indicated that there was correlation between the data and factor analysis could be performed. (2) confirmatory factor analysis (CFA) was conducted. Convergent and discriminant validity was tested by using Amos 21.0 with Maximum Likelihood Estimation. By Hair Jr. et al. (2010), the Cronbach’s alpha, composite reliability (CR), and average variance extracted (AVE) were considered as the main criteria for examining reliability and convergent validity. “Convergent validity, which examines whether individual indicators are indeed measuring the constructs they are purported to measure, was assessed using standardized indicator factor loadings, and they should be significant and exceed 0.7, and average variance extracted (AVE) by each construct should exceed the variance due to measurement error for that construct (i.e., AVE should exceed 0.50)” (Teo and van Schaik, 2012, p: 182). The results of Table 1 showed that the SFLs of all items of the constructs were above 0.7, the mean variance extraction AVE values (ranging from 0.699 to 0.810) were far higher than the threshold value of 0.50, and the composite reliability (CR) values were above 0.8, thus indicating that this measurement model in this study established the convergent validity of all the measurement items. Besides, Table 2 indicated that the square root of AVE (shown in parentheses along the diagonal) of each construct was higher (0.836 to 0.900) than corresponding correlation values for that variable in all cases, thereby assuring discriminant validity. Through the above analysis, the corresponding structure between the observation index and the item of the questionnaire was reasonable, which can accurately reflect the real situation of students’ online learning.

**Table 2:** Variable correlations and the discriminant validity for the measurement model.

Dimensions	Variables	OE	RS	OI	OC	RA	SE	LM	VI	SR
Environment	OE	(0.881)								
	RS	0.787**	(0.875)							
Behavior	OI	0.694**	0.741**	(0.860)						
	OC	0.684**	0.710**	0.769**	(0.889)					
	RA	0.597**	0.632**	0.583**	0.613**	(0.878)				
Person	SE	0.499**	0.529**	0.536**	0.571**	0.584**	(0.836)			
	LM	0.423**	0.461**	0.505**	0.554**	0.452**	0.576**	(0.900)		
Empowerment	VI	0.797**	0.621**	0.625**	0.581**	0.549**	0.465**	0.415**	(0.871)	
	SR	0.639**	0.662**	0.751**	0.798**	0.614**	0.614**	0.565**	0.572**	(0.895)

N = 396. **p < 0.01. Diagonal in parentheses: square root of average variance extracted from observed variables (items); and off-diagonal: correlations between constructs.

### The analysis of two-way correlations among the variables

Pearson’s correlation matrices for the relations between variables which were displayed in Table 2 indicated that there were significant correlations among the study variables. For example, there was a strong correlation between OE and VI (*r* = 0.797, *p* < 0.01), OC and SR (*r* = 0.798, *p* < 0.01), and a moderate correlation between SE and SR (*r* = 0.614, *p* < 0.01). These results showed that there was a correlation between learner empowerment and the influencing factors. But none of the correlation coefficients exceeded 0.80, excluding the issue of multicollinearity (Tabachnick and Fidell, 2007). These results supported the research hypotheses of this study. To further examine the research hypotheses, the following model analyses were conducted to be linked with the above correlations of variables. Once the set of objects distinguishes from another variable, discriminate validity is recognized (Fornell and Larcker, 1981; Hair et al., 2019). Therefore, Fornell–Larcker criteria (1981) suggested a correlational matrix. According to the Fornell and Larcker (1981) test proposed, the values within the diagonal of all construct denotes square roots of AVE must be greater than its column and row, which is the correlation among constructs, and each AVEs of constructs surpassed 0.5. Again, the correlations among the variables were less than 0.90, as depicted in Table 2. Therefore, these results also meet the recommended guidelines regarding discriminant validity.

### Path analysis and hypothesis verification

**Figure 2:**
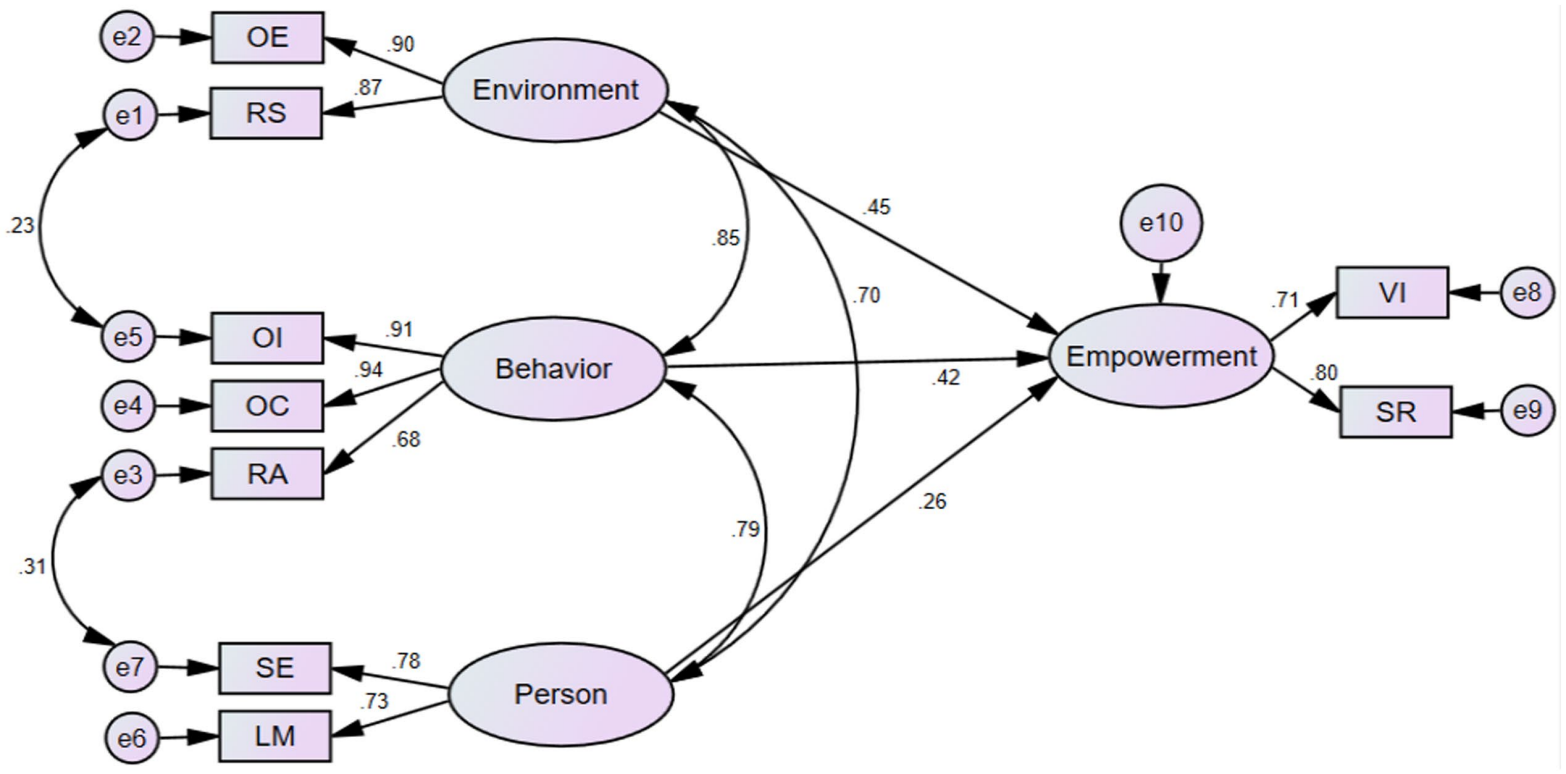
Final research model.

This study adopted Amos 21.1.0 to test and analyze the hypothesis model of influencing factors of learners’ empowerment. The test result of the initial research hypothesis model (Figure 1) concerning the structure of the three latent variables influencing learners’ empowerment manifested unsatisfactory fitting values with X^2^/df = 3.426, TLI = 0.925, CFI = 0.926, RMSEA = 0.083, SRMR = 0.071. According to the modification indices in AMOS 21.0, the structure equation model can be well established by adding corresponding paths. This study added the path between e1 and e5, and the path between e3 and e7 (the M.I. values of these two paths were 31.212 and 20.732, respectively, indicating that there are correlations between resource support and online interaction behavior, resource acquisition, and self-evaluation). Consequently, after adding the two paths, the modified structural model (Figure 2) achieved a better fit (X^2^/df = 2.815 < 3, TLI = 0.977 > 0.9, CFI = 0.987 > 0.9, RMSEA = 0.068 < 0.08, SRMR = 0.056 < 0.08), the results indicated that the model had a good fit with the observed data. The final research model and standardized estimates were shown in Figure 2.

In the inner model, the dimensions of environment, behavior, person, and empowerment were taken as formative indicators for the second-order latent variable. “The main advantage of a repeated indicator approach was that it took all constructs into consideration simultaneously, instead of measuring the second-order and first-order constructs independently.” (Tehseen et al., 2019, p: 96) Based on the final research model (Figure 2), the results showed that: (1) the item parameter estimates revealed that online environment (OE) and resource support (RS) were significant in explaining environment elements, with the online environment (OE) having the largest parameter estimate (*r* = 0.904, *p* < 0.001), which better reflects the students’ needs for the good construction of online environment in the process of online learning; (2) in the dimension of online behaviors, the item parameter estimates of online interaction (OI) and online collaboration (OC) were all greater than 0.9, with the online collaboration having the largest parameter estimate (*r* = 0.936, *p* < 0.001), indicating that online collaboration can better reflect the expectation of students in the process of online learning; (3) in the dimension of person factors, the item parameter estimates were also greater than 0.7, which indicated that during online learning students entailed stronger self-evaluation (SE) and learning motivation (LM); (4) in the dimension of learners’ empowerment, the item parameter estimates of value identification (VI) and self-reinforcement (SR) were both more than 0.7 (*p* < 0.001), which could reflect the students’ subjective empowerment in online learning. The summary of model parameter estimates and the reliability and validity results were shown in Table 3.

**Table 3:** Summary of model parameter estimates and the reliability and validity results.

Measurement indicators	Factor loadings	S.E.	t-value	R^2^	CR	AVE
OE	0.904	0.050	22.665	0.819	0.881	0.787
RS	0.870	—	—	0.756		
OI	0.914	0.091	16.055	0.837	0.886	0.725
OC	0.936	0.092	16.316	0.875		
RA	0.681	—	—	0.456		
SE	0.776	0.078	12.862	0.602	0.722	0.565
LM	0.727	—	—	0.529		
VI	0.711	—	—	0.506	0.730	0.576
SR	0.804	0.069	15.662	0.647		

—This value was fixed at 1.00 for model identification purposes.

Besides, the results of research model testing indicated that the 2 variables (OE and RS) explained 89% of the variance in environment elements; the 3 variables (OI, OC, and RA) explained 53% of the variance in online behaviors; and the 2 variables (SE and LM) explained 87% of the variance in person factors.

Based on the process of model fitting, the model fitting degree was better when the three dimensions in the reciprocal determinism established the relation with each other, which reflected the data-level correlation among the three dimensions. Therefore, bivariate correlation analysis was used to verify the positive–negative correlation and the strong-weak correlation of the three dimensions of reciprocal determinism (Table 4). The results showed that the Pearson’s correlation coefficient between environmental factors and online behavior was 0.782, and in the same way, environment elements and person factors (*r* = 0.664), and online behaviors and person factors (*r* = 0.562) also had a strong correlation, and the value of each dimension was less than 0.01 in significance of positive correlation. In addition, Figure 2 demonstrates that the estimates of correlations among exogenous variables of environment elements, online behaviors, and person factors were 0.854, 0.793, and 0.700, respectively, and the extremely significant *p* value was less than 0.001. Through the above analysis, it indicated that when any dimension in the reciprocal determinism was in high demand, it will cause the other two dimensions to make corresponding changes.

**Table 4:** The correlation hypothesis verification of the three dimensions of reciprocal determinism.

Variable relations	Correlations among exogenous variables in structure model	Pearson correlations	Results
Environment↔Behavior	0.854***	0.782**	Supporting H1
Behavior↔Person	0.793***	0.664**	Supporting H2
Environment↔Person	0.700***	0.562**	Supporting H3

***p < 0.001; **p < 0.01.

Based on the analysis of fitting paths, it can be found that: (1) there was a direct positive relationship between environment elements and learners’ empowerment, with the path coefficient of 0.447 (*p* < 0.001). There was a direct and positive relationship between online behaviors and learners’ empowerment, with a path coefficient of 0.423 (*p* < 0.001), and a direct and positive relationship between person factors and learners’ empowerment, with a path coefficient of 0.262 (*p* < 0.001). (2) Through the analysis of standardized influence effects, the degree of effect on learners’ empowerment was: environment elements (*β* = 0.447) > online behaviors (*β* = 0.423) > person factors (*β* = 0.262). The results are shown in Table 5.

**Table 5:** The relationship coefficients of related path and the hypothesis verification.

Path relations	Pearson correlations	Standardized path coefficients	Results
Environment→Empowerment	0.712**	0.447***	Supporting H4
Behavior→Empowerment	0.798**	0.423***	Supporting H5
Person→Empowerment	0.699**	0.262***	Supporting H6

***p < 0.001; **p < 0.01.

## Discussion

In terms of environment elements, the standardized correlation weight for online environment and resource support was *r* = 0.904 and *r* = 0.870, respectively, indicating that the online environment is the determinant for students’ perceiving of environment elements. This pattern of finding is consistent with that already depicted in the previous literature (Clayton et al., 2010) which indicated that those students who preferred learning environments incorporating an online component showed significantly more of a mastery of resource orientation and greater interest in obtaining resource support than those students who preferred the traditional face-to-face classroom instruction. Of the observed measures of online behaviors, the standardized correlation weight for online interaction (*r* = 0.914) and online collaboration (*r* = 0.936) was great, which showed that online interaction and online cooperation constituted the key components for students to perceive of online behaviors, and when students perceived of online learning being characterized by clear goals and well-organized interpersonal interactions, their behavioral engagement in online learning would also be enhanced. This is in line with previous research which indicated that an orderly classroom could improve learners’ engagement and deepen their understanding and cognition of the nature of language (Devlin and Samarawickrema, 2010), and that in-depth teacher-student/student–student interactions could help learners form positive emotional experience and engagement behaviors (Loewen and Sato, 2018). This finding also highlighted the significance of constructing the flexibility of online environments, as Houlden and Veletsianos (2019) addressed that “flexibility stands to make education more student-centered, empowering learners to make choices that align with their needs and interests, potentially leading to greater engagement with, participation in and completion of their studies” (p: 1007). In the measurement model of individual factors, both self-evaluation and learning motivation have great contribution to person factors, which is similar to the previous research that considered self-evaluation and learning motivation as important internal motivational index to promote students’ online learning behaviors. Besides, this study found that self-evaluation, as an individual element, contributed more to and determined learners’ empowerment. The finding is aligned with that already reported in the literature (Kilis and Yıldırım, 2018; Yang et al., 2020) which claimed that self-evaluation ability can affect the social presence, improve the social motivation and strategy of the individual, influence the value identity of the learner, and ultimately affect learners’ online learning experience and self-reinforcement tendency.

In addition, there were significant correlations between value identification and self-reinforcement in learners’ empowerment and the 7 observed indicators of the other three latent variable dimensions as well. The online environment was the main influencing factor of value identification, and the online interaction and online collaboration were the main influencing factors of self-reinforcement. The effect of online environment, online interaction, and online collaboration on value identification and self-reinforcement indicated that the effective organization of online environment and online learning activities had the largest impact on learners’ empowerment, and meanwhile, it reflected that learners’ empowerment was more significantly affected by the organizational structure of the teaching environment. The results also showed that students’ information literacy and sensitivity to the online learning environment also supported their active participation in online learning, and achieved a better result of learners’ empowerment.

This study evidenced that the three dimensions of reciprocal determinism had strong positive correlations. In other words, high demand in any one dimension would lead to changes in the content of the other two dimensions, which is consistent with Bandura (2001)’s definition of the relationship between the three, namely significant interrelationships among “environment elements,” “online behaviors” and “person factors.” In view of the fact that previous studies paid too much attention to the influence of person factors on learning behaviors, this finding is particularly important, for it indicates that in actual online learning, learning behavior is not only restricted by learners’ individual subjective factors, but also affected by the external environment and social norms, with both interweaving and working together. This finding also corroborated previous research, verifying that a good form of classroom organization helped maintain teaching activities and prepare students for classroom learning (Hennessy et al., 2021); and that both external environmental elements and motivation components can affect students’ learning behaviors (Herzberg and Mausner, 1993) and stimulate learning initiative. In addition, the three latent variables (i.g. environment, behavior, person) also had a positive direct influence on the effect of learners’ empowerment, which can be used to predict learners’ empowerment directly. These findings are consistent with the research of Yang et al. (2020) that teaching environments and individual cognitive presence were important predictors of learners’ empowerment in online learning. In the influence intensity, the effect relation of the three latent variables on learners’ empowerment is environment elements > online behaviors > person factors. It also proved that the effective classroom environment and supportive system design conduced to improving the level of foreign language learners’ behavioral engagement, which is in line with the conclusions of previous studies. For instance, through empirical research, Rowe et al. (2010) found that adequate teaching preparation and harmonious classroom atmosphere contributed to the establishment of appropriate teaching situations and the improvement of students’ classroom participation; Christenson et al. (2012) showed that a supportive institutional environment had a positive effect on the formation of sustained behavioral engagement.

### Implications and limitations

Based on the results of the analysis and the presentation of the final model, the following implications can be drawn. Firstly, from the results of the study on the impact of environment factors on learners’ empowerment, the present study asserted that we need to examine the rationality of the structural design of online learning environments. For students, online learning has a certain challenge and is sometimes problematic as it differs from traditional classroom learning and thus students suffer from a certain degree of maladaptation (Mamun et al., 2020). But this kind of maladaptation can feed back to the rationality of online environment design directly. Therefore, how to realize the organic combination of online learning environment with the overall instructional design, and how to improve the adaptability of students are worth taking into account. For instance, in the allocation of learning resources, it is imperative to conceive of the differential needs of students for learning resources, develop school-based learning resources on the basis of rational use of existing learning resources, and promote students’ value identification with school-based culture. Teachers and educational institutions should change their roles under the traditional teaching environment and fully understand students’ individual characteristics, which emphasizes the student-centered concept of helping students learn to understand the use, cognition, selection, and reconstruction of information, so as to complete the construction of knowledge (Lai, 2015).

Secondly, grounded on the results of the study on the impact of online behaviors on learners’ empowerment, this study suggested that attention should be paid to the interaction and collaboration between teachers and students in online learning. Teacher-student interaction, as a common way of communication in online learning, is an important medium for teachers to convey knowledge to students and help students to accept and reconstruct knowledge, as the effectiveness of the interaction directly affects the improvement of students’ learning effect (Lai et al., 2012). Therefore, this study expanded the existing literature regarding teacher-student interaction and collaboration in online learning, by highlighting teacher-student communication as result-oriented, interactive functions optimized, resources personalized, interaction with resources prolonged, and artificial intelligence and technologies integrated to enhance the students’ sense of online presence.

Thirdly, in view of the influence of person factors on learners’ empowerment, this study advocated that more concerns should be given to the development of student autonomy under the condition of online learning, especially for Chinese students who were influenced by traditional Chinese teaching culture of that “students are the indoctrinated subject in the teaching process, and they are subordinate in the teaching activities” (Zimmerman and Schunk, 2011, p: 10). In the process of online learning, the separation of online time and space resulted in the weakening of students’ learning autonomy and the widening communication drawbacks between teachers and students. Thus, it is the key to scaffold the idea of student autonomy and promote students’ self-evaluation and learning motivation. The study supported the argument that individual espoused orientation factors (e.g., self-evaluation) do influence language learners’ empowerment for learning online. This study thus added to our understanding of how to elevate learners’ internal attribution, such as guiding students to make clear the goal of autonomous learning, assisting students programming the path of self-directed learning, providing necessary learning scaffolds, and choosing appropriate ways to intervene learning difficulties. To some extent, students’ learning autonomy reflects the initiative, enthusiasm, and consciousness of creating an active learning environment. Thus, this study further highlighted to strengthen the self-regulation of students’ motivational consciousness and give full play to students’ subjective initiative, so as to improve the level of students’ self-determination. In a word, students’ learning autonomy is an internal habit, and the cultivation of the consciousness of autonomy, motivation, and challenge needs the assistance of external environments and effective teacher-student interactive communication, thus the coordination mechanism between internal and external factors needs to be optimized.

Despite this study adopted rigorous testing procedure, some limitations existed. First, this research relied on students’ self-reported data, which may have affected the accuracy of the results. Second, this study only investigated some of the influencing factors of online learning supporting learners’ empowerment, thus a certain degree of one-sidedness may occur. Further research can develop more scales, such as motivation and developmental assessment, and integrate qualitative research methods (e.g., interviews and classroom observation), to deeply, systematically, and comprehensively explain the influencing factors of online learning supporting learners’ empowerment and its functioning mechanism. Third, this study considered learners’ empowerment as a whole, focusing on its influencing factors and its mechanism. However, the two observed variables of learners’ empowerment in this study, namely, value identification and self-reinforcement, may be affected by comprehensive factors. Therefore, the follow-up studies can be conducted on the in-depth exploration of the different aspects of learners’ empowerment influencing factors and their impact mechanism. Fourth, this study focused on the online learning of college English course. As a foreign language, the nature of the subject may affect the learners’ empowerment and learning experiences; therefore, the application of the results of the study to other subjects still should be cautious.

## Conclusion

At present, online learning is frequently promoted as a flexible approach to education. In this educational landscape, this study examined the contribution of environment elements, online behaviors, and person factors to learners’ empowerment in a sample of Chinese undergraduate students. The analysis of fitting results did find that, in the online learning context, learners’ empowerment was positively and significantly impacted by environment elements, online behaviors, and person factors. Thus, it helps educational authorities and schools understand the specific contribution of external and internal factors to learners’ empowerment of using technology for online learning and informs the construction of sufficient technology and resource support to help students to overcome potential maladaptation to online learning, as well as choosing online learning platforms that have been customized. This study further identified online interaction, online orientation, and resource acquisition, as critical to learners’ online behaviors in online learning supporting learners’ empowerment. This suggests the importance of focusing on providing sufficient guidance and more online collaborative opportunity for university students’ online learning. On the whole, the study not only has the important enlightenment to the technology-driven educational practice, but also provides a new perspective for researchers to better understand learners’ empowerment and its associations with students’ self-regulated learning capability and online learning community.

## Data availability statement

The original contributions presented in the study are included in the article/supplementary material, further inquiries can be directed to the corresponding author.

## Ethics statement

Ethical review and approval were not required for the study on human participants in accordance with the local legislation and institutional requirements. Written informed consent for participation was not required for this study in accordance with the national legislation and the institutional requirements.

## Author contributions

The author confirms being the sole contributor of this work and has approved it for publication.

## Funding

This work was supported by the Research Project of the 2021 Teaching Reform of Xingzhi College, Zhejiang Normal University (Grant No. ZC303921073).

## Conflict of interest

The author declares that the research was conducted in the absence of any commercial or financial relationships that could be construed as a potential conflict of interest.

## Publisher’s note

All claims expressed in this article are solely those of the authors and do not necessarily represent those of their affiliated organizations, or those of the publisher, the editors and the reviewers. Any product that may be evaluated in this article, or claim that may be made by its manufacturer, is not guaranteed or endorsed by the publisher.
